# The complete chloroplast genome of *Callianthe picta* (Malvaceae)

**DOI:** 10.1080/23802359.2021.2005487

**Published:** 2021-12-10

**Authors:** Sujie Wang, Shujin Ding, Ruyou Deng, Wanyuan Shi, Hanyao Zhang

**Affiliations:** a Key Laboratory for Forest Resources Conservation and Utilization in the Southwest Mountains of China Ministry of Education, Southwest Forestry University, Kunming, China; b Key Laboratory of Biodiversity Conservation in Southwest China, National Forest and Glassland Administration, Southwest Forestry University, Kunming, China

**Keywords:** *Callianthe picta*, chloroplast genome, phylogenetic analysis

## Abstract

*Callianthe picta* likes a warm and humid climate, is resistant to barrenness, and is easy to reproduce. Its petals and leaves can promote blood circulation and remove blood stasis, and can also be used to relax the muscles and collaterals. In this study, we sequenced the complete chloroplast genome sequence of *C. picta* to investigate its phylogenetic relationship in the family Abutilon. The complete chloroplast size of *C. picta* is 160,398 bp, including a large single-copy (LSC) region of 89,088 bp, a small single-copy (SSC) region of 20,138 bp, a pair of invert repeats (IRs) regions of 25,586 bp. The GC content of the whole complete chloroplast genome is 37.0%. We annotated 128 genes in the genome in detail, including 84 protein-coding genes, 36 tRNA genes, and 8 rRNA genes. Phylogenetic analysis indicated that *C. picta* was closely related to *Abutilon theophrati*.

*Callianthe picta* (Gillies ex Hook. & Arn.) Donnell 1834 known as *Sida striata* is a kind of evergreen shrub of the mallow family Abutilon, mainly native to Brazil, Uruguay and other places in South America, Southwest of China and other parts of the region also have introduced cultivation (Jiang and Jiang 2005). Besides being ornamental, petals and leaves of *C. picta* play a significant role in activating blood circulation and removing blood stasis (Peng et al. 2006). The chloroplast genomes are widely used in many research fields such as plant identification and recognition, phylogenetic analysis, and genetic diversity evaluation (Dong et al. 2018; Sun et al. 2020). In the present study, we reported the chloroplast complete genome sequence of *C. picta* for the first time and performed phylogenetic analysis to provide valuable materials and information for further in-depth study of this species.

The leaf specimens of *C. picta* were collected from Kunming, Yunnan, China (102°33′27″E, 25°7′5″N, 1928 m). A specimen was deposited at the Herbarium of Southwest Forestry University (bbg.swfu.edu.cn, Dr.Yao and bbg01@swfu.edu.cn) under the voucher number: SWFU-AAP-APT-3652. Total DNA was extracted using the CTAB method (Doyle and Doyle 1987) and sequenced on the lllumina NovaSeq 6000 platform from Annuoyouda Biotechnology Co., Ltd. (Zhejiang, China). About 5.1 GB of high-quality clean reads were generated with adaptors trimmed. Then, the complete chloroplast genome was assembled by GetOrganelle v1.6.2 (Jin et al. 2020). Genome annotation was performed by Geneious R9 (Kearse et al. 2012) and manually adjusted by comparing it to the reference chloroplast genome of *Abutilon theophrasti* (GenBank accession number NC053702). The annotated genomic sequence has been submitted to GenBank (accession number: MZ615336).

The chloroplast genome of *C. picta* is a circular DNA molecule with a length of 160,398bp, which contains a large single-copy region (LSC), a small single-copy region (SSC), and a pair of inverted repeat sequences (IRs), with lengths of 89,088, 20,138, and 25,586 bp, respectively. The content of guanine (G) and cytosine (C) in the whole chloroplast genome is 37.0%. In addition, it contains 128 genes with different functional classifications, of which 84 are functional genes encoding proteins, 36 are tRNA genes, and 8 are rRNA genes.

Available chloroplast genomic information of Abutilon plants was limited. So we selected 16 related plant species to study their phylogenetic position with *C. picta.* First, we used the Clustal W program to compare multiple sequences with default parameters, saved the comparison results, and imported them into MEGA X. We used the neighbor-joining (NJ) method to construct the evolutionary tree, with the combination of the boot Tstrap method (1000 replicates) (Kumar et al. 2018). *Arabidopsis thaliana* (NC000932) was served as the out-group. It was found that *C. picta* was more closely related to *A. theophrati* than to species from other taxonomic groups (Figure 1). The complete genome-wide determination of *C. picta* chloroplasts provides new molecular data for elucidating the evolutionary relationship of Malvaceae and other biological studies.

**Figure 1.**
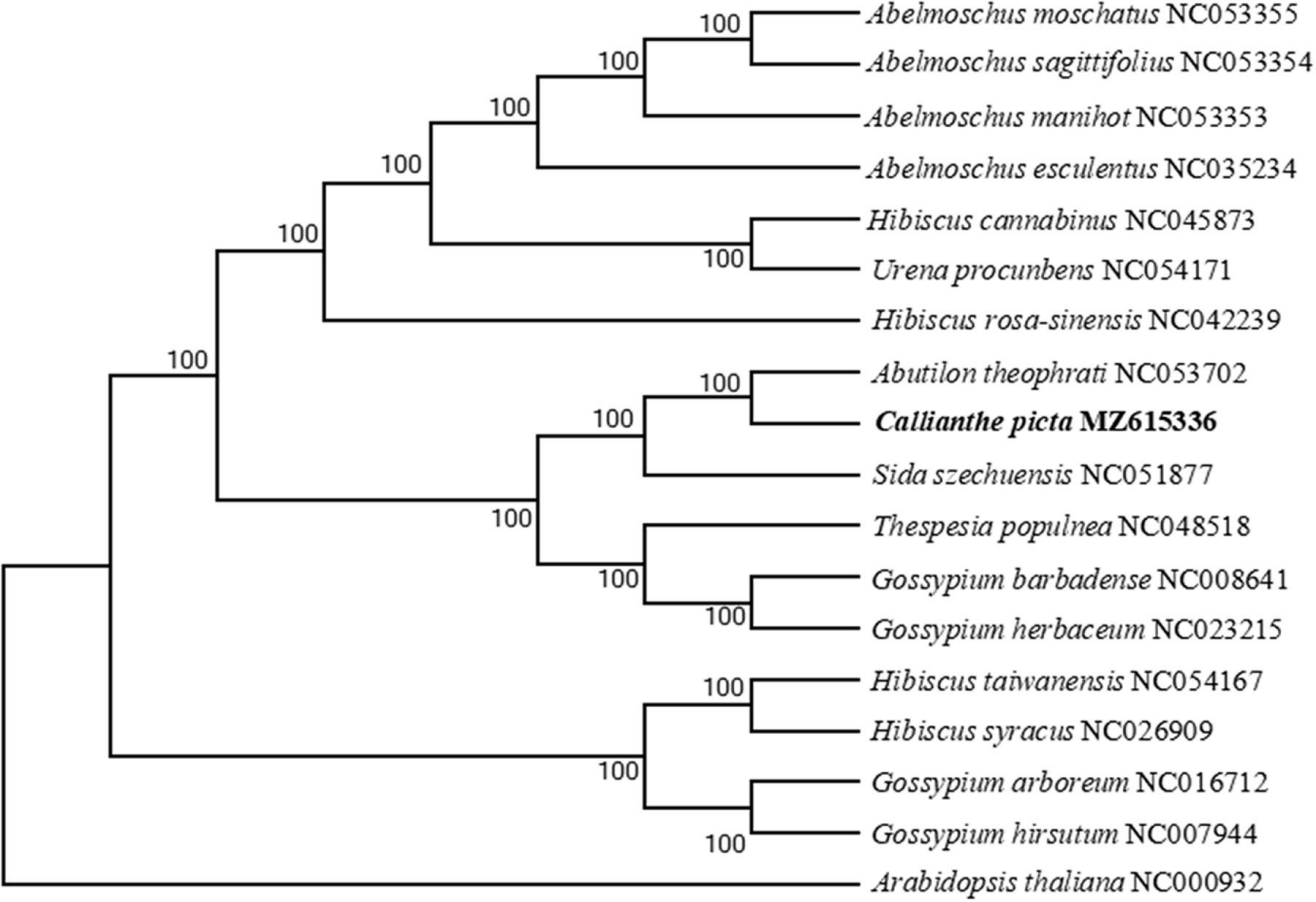
Phylogenetic tree based on seventeen complete chloroplast genome sequences. The bootstrap value based on 1000 replicates is shown on each node.

## Data Availability

The voucher specimens of *Callianthe picta* were deposited at the Herbarium of Southwest Forestry University, Kunming, Yunnan, China (accession number: SWFU-AAP-APT-3652). The genome sequence data that support the findings of this study are openly available in GenBank of NCBI at [https://www.ncbi.nlm.nih.gov] (https://www.ncbi.nlm.nih.gov/) under the accession no. MZ615336. The associated BioProject, SRA, and Bio-Sample numbers are PRJNA481636, SRR7589393, and SAMN09691266, respectively.
